# Thyroid-Stimulating Hormone: An Important Target for the Prevention of Nonalcoholic Fatty Liver Disease

**DOI:** 10.33549/physiolres.935453

**Published:** 2025-04-01

**Authors:** Zixuan WANG, Hanyu WANG, Hui SUN

**Affiliations:** 1 Department of Endocrinology, Union Hospital, Tongji Medical College, Huazhong University of Science and Technology, Wuhan, China; 2 Hubei Provincial Clinical Research Center for Diabetes and Metabolic Disorders, Wuhan, China

**Keywords:** Thyroid stimulating hormone, Subclinical hypothyroidism, Nonalcoholic fatty liver disease, Nonalcoholic steatohepatitis disease, Lipid

## Abstract

Non-alcoholic fatty liver disease (NAFLD) is one of the leading causes of cirrhosis and liver cancer. Its global prevalence increases annually, significantly affecting quality of life. Only a few patients manage to alleviate NAFLD through lifestyle modifications. The pathogenic mechanisms and therapeutic targets of this disease remain inadequately explored. In recent years, many studies highlighted a close relationship between the thyroid-stimulating hormone (TSH) and NAFLD. TSH has been shown to promote fat accumulation in the liver by participating in the ab initio synthesis, uptake and secretion of lipids. Moreover, TSH exacerbates hepatocyte inflammation and fibrosis by inducing endoplasmic reticulum stress and promoting the release of inflammatory factors. Although some of the conclusions remain controversial and are subject to debate, exploring the connection and possible pathways between TSH and NAFLD is crucial. Such research could advance early clinical prevention and intervention strategies, thereby reducing the incidence of severe NAFLD-associated comorbidities. Therefore, this review aims to summarize currently available evidence on the association between TSH and NAFLD focusing on the following objectives: elucidating the relationship between TSH and NAFLD; TSH may be a potential target for NAFLD prevention; exploring potential molecular targets that could block TSH-mediated promotion of NAFLD.

## Introduction

NAFLD is a metabolism-related liver disease, which is based on the hepatic lipid accumulation in the absence of excessive alcohol consumption [1,2]. This condition progresses through stages, including hepatic steatosis, non-alcoholic steatohepatitis (NASH), fibrosis, and in severe cases, cirrhosis. Over half of NAFLD cases are classified as NASH, which represents one of the major subtypes of liver fibrosis progressing to cirrhosis [3]. The global prevalence of NAFLD has been steadily increasing year on an annual basis. A recent meta-analysis, based on data from 1990 to 2019, estimated that NAFLD affected at least 30 % of the adult population. However, data obtained from 2016 to 2019 revealed a prevalence as high as 38 %. With the contemporary rise in obesity and metabolic diseases, the burden of NAFLD is anticipated to increase further. Mathematical models predict that between 2016 and 2030, China will experience the highest number of deaths from liver-related diseases, while the incidence of decompensated cirrhosis in the United States is projected to increase by 1.5-fold [4–8].

Hypothyroidism, both subclinical and overt, is a growing global health concern with an increasing incidence. Numerous studies have demonstrated a correlation between thyroid hormones (triiodothyronine (T3) and thyroxin (T4)) and the severity of NAFLD in overt hypothyroidism. Hypothyroidism is associated with elevated serum triglyceride and cholesterol levels, which contribute to the risk of developing NAFLD. Patients with hypothyroidism have a higher risk of NAFLD, with subclinical hypothyroidism being characterized by elevated serum TSH levels, while serum-free thyroxin remains within normal reference ranges. Serum TSH concentration is considered the most sensitive and specific indicator for screening thyroid function and assessing early thyroid metabolic status. Recent findings suggest that even in subclinical hypothyroidism (SCH), where thyroid hormone levels are normal, elevated TSH levels may influence the progression of NAFLD [9–13]. It’s important to note that the prevalence of this subgroup of patients is increasing annually.

A study conducted in the United States reported that the prevalence of overt and subclinical hypothyroidism was 0.4 % and 9 %, respectively. Moreover, subclinical hypothyroidism was found in more than 20 % of women aged 75 years or older. Similarly, a meta-analysis conducted in Europe revealed a prevalence of 0.37 % for overt and subclinical and 3.8 % for hypothyroidism [14]. To date, there is no consensus regarding the benefits of early thyroid hormone replacement therapy in preventing and minimizing the development and progression of NAFLD [15]. Understanding the mechanistic pathways linking the preclinical stage of thyroid dysfunction and NAFLD could provide critical insights and serve as a foundation for the development of early prevention and intervention strategies for NAFLD.

## Relationship between TSH and NAFLD

Some studies have suggested a significant association between TSH and NAFLD. TSH can directly or indirectly affect NAFLD. A systematic review that involved 42,227 participants revealed a significant association between elevated TSH levels and NAFLD [16]. Presley H. Nichols *et al*. employed causal mediation analysis to further demonstrate that the effects of TSH on NAFLD are not related to obesity [17]. This suggests an independent association between serum TSH levels and NAFLD. Another cross-sectional study found that hepatitis B patients with high levels of TSH exhibited a higher prevalence of NASH, an earlier onset of the disease, and more severe hepatic steatosis compared to those with normal TSH levels. The study also identified a positive correlation between TSH levels and the prevalence of NASH, unaffected by confounding factors such as the body mass index (BMI) and metabolic syndrome [18].

A study that used magnetic resonance imaging (MRI) techniques to precisely quantify hepatic fat content found that, among non-obese individuals, serum TSH levels were positively correlated with hepatic fat deposition [19]. TSH has also been linked to late-stage hepatic fibrosis. A meta-analysis indicated that TSH was significantly associated with advanced liver fibrosis [20]. Prospective cohort studies have also found a positive association between high TSH levels and the risk of NAFLD combined with liver stiffness measurements of ≥8.0 kPa [21]. Collectively, these findings suggest that TSH levels could serve as a valuable biomarker for evaluating the progression of liver fibrosis in patients with NAFLD.

Although the mechanism through which TSH contributes to the development of NAFLD has not been clarified, several studies suggest its indirect involvement via lipid metabolism, especially in elderly populations [22]. NAFLD has been linked to significant alterations in the liver and serum lipidome during steatosis [23]. TSH has been shown to influence lipid metabolism, with evidence indicating that SCH is associated with a higher propensity for postprandial hyperlipidemia compared to individuals with normal thyroid function. Tanaci *et al*. demonstrated that triglyceride (TG) levels were notably higher in the SCH population and individuals with TSH levels above 5 mIU/L were seven times more likely to develop postprandial hyperlipidemia compared to the general population, demonstrating TSH’s effects on TG metabolism [24].

Studies have found an increased risk of overweight and obesity among adolescent females with higher serum TSH levels [25]. Cross-sectional studies further demonstrated the presence of a linear relationship between TSH levels within the reference range and lipid profile [26]. In addition, a retrospective study identified SCH and TSH levels as the sole determinant of NASH and alanine aminotransferase (ALT) levels in obese children and adolescents, independent of obesity severity [27]. Although these studies lack histological confirmation, the findings suggest that TSH indirectly influences NAFLD by affecting lipid or liver metabolism, independent of other metabolic factors such as obesity and BMI.

## Possible mechanisms of TSH in NAFLD

Although the precise pathogenic pathway linking thyrotropin and NAFLD has not yet been clarified, numerous studies have explored the potential role of TSH in the pathophysiological mechanisms underlying NAFLD. These investigations have focused on molecules associated with NAFLD development, such as AMP-activated protein kinase (AMPK)-sterol regulatory element binding protein (SREBP), leptin, and secreted phosphoprotein-1 (SPP1). From different perspectives, including lipid metabolism, fat accumulation, inflammatory infiltration, and fibrotic processes, these studies have provided insights into the possible mechanisms through which TSH may affect the development of NAFLD.

### SREBP

**Fig. 1:**
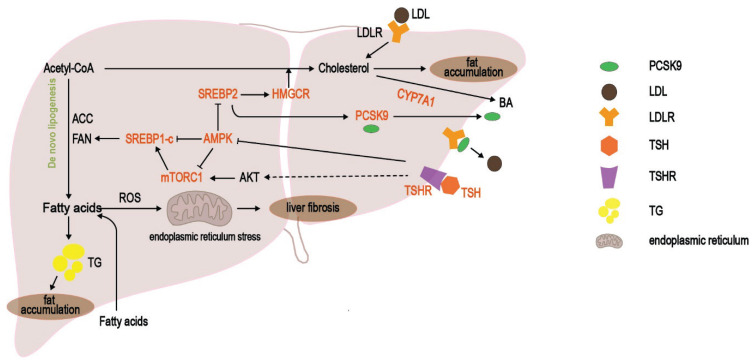
TSH exacerbates hepatic fat accumulation and fibrosis via the SREBP pathway. TSH activates SREBP to regulate the molecular activities of PCSK9, HMGCR, and CYP7A1 through the mTORC or AMPK pathways. These pathways are involved in lipid metabolism, such as fatty acid synthesis, cholesterol absorption, and bile acid synthesis and secretion, which promote hepatic fat accumulation and the development of NAFLD. In addition, elevated levels of FFAs increase the production of reactive oxygen species (ROS) promote endoplasmic reticulum stress, and promote liver fibrosis.

Lipid overaccumulation in hepatocytes is one of the major causes of NAFLD development [28]. SREBP is key transcription factor that activate cholesterol uptake, as well as fatty acid and triglyceride synthesis. These proteins are classified into three isoforms, which are, SREBP-1a, SREBP-1c, and SREBP-2. SREBP-1a exhibits the broadest activation spectrum, influencing the expression of all lipid synthesis-related downstream response genes. SREBP-1c and SREBP-2 preferentially activate fatty acid and cholesterol synthesis-related genes, respectively. The activation of SREBP1c and SREBP-2 leads to the accumulation of triglycerides and cholesterol, thereby exacerbating hepatic steatosis. Consequently, these pathways are now regarded as important therapeutic targets for managing hepatic steatosis [29,30]. TSH can activate classical de novo lipogenesis and cholesterogenic pathways through the SREBP pathways. Possible pathways include AMPK, mechanistic target of rapamycin complex (mTORC), Proprotein convertase subtilisin/kexin-9 (PCSK9), 3-hydroxy-3-methylglutaryl coenzyme A reductase (HMGCR), cholesterol 7*α*-hydroxylase (CYP7A1), and carbohydrate response element binding protein (ChREBP), which play crucial roles in lipid synthesis and transport processes (Fig. 1).

#### AMPK and mTORC

AMPK, mechanistic target of rapamycin complex 1 (mTORC1), and mechanistic target of rapamycin complex 2 (mTORC2) have been identified as upstream effector molecules of SREBP1c. mTORC2 promotes the phosphorylation of AKT, thereby activating it. AKT, also known as Protein kinase B, is a serine-threonine kinase that, when activated, can directly or indirectly activate mTORC1, leading to the subsequent activation of SREBP1c. AMPK has been recognized as a potential therapeutic target for NAFLD. Activated AMPK down-regulates the mRNA and protein expression levels of REBP-1c, although the underlying mechanism is complex. Current findings suggest that the activation of AMPK directly inhibits mTORC1 and attenuates the expression of SREBP-1c, a downstream molecule in the AKT/mTORC1 pathway. This inhibition restricts the transcription and translation of lipid synthesis-related genes such as fatty acid synthase (FASN) and acetyl-CoA carboxylase (ACC), thereby suppressing de novo lipogenesis. As a result, the lipid accumulation characteristic of hepatic steatosis is mitigated [31–33]. This represents one of the possible mechanisms. TSH has been shown to increase lipid accumulation by inhibiting key metabolic processes. It was found that in the context of NAFLD, TSH mediates its effects via the hepatic TSH receptor, thereby activating the cyclic adenosine monophosphate (cAMP)/protein kinase A (PKA) pathway, which is associated with reduced AMPK activity. This reduction in AMPK activity enhances the activation of SREBP-1c, leading to lower triglyceride metabolism. Consequently, these changes increase the accumulation of extra fat in hepatocytes, thereby enhancing the development and progression of NAFLD [34].

TSH upregulates the phosphatidylinositol 3-kinase (PI3K)/AKT pathway, which can contribute to tumor development [35]. Further studies have proven that, in cancer cells, the PI3K/AKT signaling pathway promotes the expression of ATP citrate lyase (ACL), ACC, and FASN by upregulating SREBP-1. This signaling cascade promotes fatty acid synthesis and increases low-density lipoprotein receptor (LDLR) expression, thereby facilitating cholesterol uptake [36]. These findings suggest that, under pathological conditions, TSH upregulates SREBP-1 through a PI3K/AKT-mediated pathway, potentially contributing to the pathogenesis of NAFLD. However, the intra-tumor environment is complex, and this process may be influenced by multiple factors. Whether this pathway operates similarly in the context of SCH remains unclear and warrants further investigation.

#### PCSK9 and HMGCR

PCSK9 and HMGCR are downstream response molecules of SREBP2. PCSK9, a secretory serine protease, is primarily synthesized by the liver [37] and subsequently secreted into plasma. In circulation, it binds to the LDLR, promoting its degradation. It is secreted into plasma, where binds to LDLR and promotes its degradation [38]. Animal experiments have found that PCSK9 has a positive association with non-alcohol-associated fatty liver (NAFL) and a negative association with NASH. Clinical evidence further highlighted a positive association between circulating PCSK9 levels, liver fat accumulation, and the severity of hepatic steatosis [39]. Both PCSK9 and LDLR, are modulated by SREBP-2 [40].

TSH stimulates hepatocytes to increase PCSK9 gene expression and protein levels by activating SREBP, leading to a reduction in cell surface LDLR, and diminished uptake of low-density lipoprotein cholesterol (LDL-c) by hepatocytes [41]. It is well established that the expression of HMGCR, a key enzyme in cholesterol biosynthesis, increases in NAFLD [42]. TSH has been found to upregulate both the precursor and nuclear active forms of SREBP-2, along with the target genes of HMGCR and3-hydroxy-3-methylglutaryl-coenzyme A synthase (HMGCS) [43]. Additionally, TSH activates the cAMP/PKA/CREB(cAMP-responsive element binding) signaling pathway, which mediates the transcriptional activation of HMGCR [44].

TSH plays a significant role in the progression of NAFLD by regulating the key molecule, SREBP. This is achieved through two pathways, PCSK9 or HMGCR, which regulate LDL uptake into the liver, along with lipid synthesis. However, further research is still needed to explore the specific molecular regulatory mechanisms within these pathways and to clearly define the influence of other contributing factors.

#### HNF-4α/CYP7A1

CYP7A1 is the rate-limiting enzyme in bile acid (BA) synthesis [45]. Hepatocyte nuclear factor 4 alpha (HNF-4α) is the most abundant orphan nuclear receptor expressed in the liver and plays a critical role in the regulation of CYP7A [46]. Experimental studies in rat models indicate that the NASH group had higher bile and CYP7A1 expression in the liver compared to healthy controls [47]. TSH inhibits hepatic bile acid synthesis through the SREBP-2/HNF-4α/CYP7A1 signaling pathway, independent of thyroid hormone (TH) [45,48]. This highlights the role of TSH in bile acid homeostasis, irrespective of thyroid hormone effects. Therefore, correcting TSH levels in hypothyroid patients is important in maintaining BA homeostasis. Bile acid synthesis is the main route for eliminating excess cholesterol from the body and maintaining the homeostasis of cholesterol metabolism [48]. Accumulation of free cholesterol in the liver promotes the development of NAFLD [42]. Zeyu Dong *et al*. found that hepatic cholesterol-25-hydroxylase (Ch25h) inhibition attenuated CYP7A1-dependent bile acid biosynthesis and secretion, thereby exacerbating hepatic steatosis, at the onset of NAFLD in mice fed a high-fat diet [49]. TSH affects the metabolic homeostasis of hepatic bile acids through the aforementioned signaling pathway, which, in turn, affects the progression of NAFLD. However, further clinical and basic research is necessary to clarify whether this sequential process operates entirely independent of thyroid hormones or whether TSH directly contributes to the pathogenesis of NAFLD through this signaling pathway.

### ChREBP

ChREBP (Carbohydrate response element binding protein) binds to the carbohydrate response element in the promoter of lipogenic genes. This upregulates the transcriptional activity of related genes and promotes de novo lipogenesis (DNL) in the liver. As a prime factor of DNL, which is an important fatty acid source in NAFLD, ChREBP significantly contributes to lipid accumulation [50]. Higher levels of ChREBP protein have been observed in NASH patients compared to individuals with healthy liver [51]. Furthermore, SREBP-1c and ChREBP exhibit reciprocal regulatory interactions [52]. In a study that involved hypothyroid rats treated with the T3 peripheral metabolite T2, it was found that T2 reduced the hepatic mRNA level of SREBP-1 while increasing the circulatory free fatty acids (FFA) and ChREBP content, thereby mitigating hyperlipidemia [53]. As discussed earlier, TSH activates the SREBP pathway. It is reasonable to suppose that TSH can be involved in the pathogenesis of NAFLD by inducing ChREBP expression mediated by SREBP. However, critical details still lack support from scientific experiments. This includes the specific molecules and pathways involved in the interaction between the two, their precise role in lipid metabolism aspects, and how to exclude the effect of ChREBP on SREBP-1c to validate that TSH is indeed involved in the processes regulated by ChREBP.

### Leptin

Leptin is a molecule that is primarily secreted by adipose tissue and is involved in regulating metabolism and energy homeostasis. Studies have reported that leptin is linked to the development of NAFLD [54]. Serum leptin levels are positively associated with the severity of liver inflammation and fibrosis [55]. Experimental studies have proven that in advanced stages of NAFLD, leptin may act as an inflammatory and fibrogenic factor [56]. Clinical studies have found that levels are associated with greater disease severity [57]. Cohort studies have shown that leptin can be a promising indicator for NAFLD diagnosis, with minimal influence from BMI. A leptin threshold of >9.33 ng/ml demonstrated significant sensitivity and specificity for NAFLD diagnosis [58]. Ferruccio Santini et al. reported that acute recombinant human TSH (rhTSH) significantly increased serum leptin concentrations in vivo and demonstrated the existence of a functional thyrotropin receptor (TSHR) on the surface of human adipocytes in vivo, as well as the role of TSH in controlling leptin secretion [59]. This finding aligns with Aysin Oge’s observation of a correlation between serum leptin levels and TSH in hypothyroidism [60]. This relationship was further confirmed by subsequent studies, which showed that higher TSH levels were associated with higher serum leptin levels [61,62]. More importantly, Aysin Oge et al demonstrated that the correlation between leptin and TSH was independent of the TH [60]. However, the underlying mechanisms of the interactions between the relevant metabolic factors such as TSH, leptin, and obesity remain unclear. While some studies highlight a disease- or population-specific relationship between leptin and TSH [63], others report no significant association [64]. Moreover, the current study lacks corresponding basic molecular studies that explore these interactions, thereby necessitating further investigation.

### FASN and FFA

FASN is a key enzyme in adipogenesis and an important contributor to the progression of many metabolic disorders such as cancer, obesity, and NAFLD [65–67]. Its expression is higher in patients with NAFLD [68]. Evidence from existing studies indicates that FASN promotes the development of NAFLD by regulating hepatic lipid metabolism. Inhibition of FASN activity reduces the severity of NAFLD [69,70]. While TSH may regulate FASN through the SREBP pathway, there is a lack of relevant studies on whether TSH can affect lipid accumulation in the liver via FASN. The results of cellular studies and animal experiments are also inconsistent. In vitro studies showed that the inhibition of PKA and protein kinase RNA-like endoplasmic reticulum kinase (PERK) pathways downregulates TSH-TSHR-induced inhibition of FASN protein and mRNA expression, correlating with the previously discussed activation of PKA by TSH. However, in vivo studies present a contrasting scenario: TSHR expression was significantly increased in visceral tissue cells of high-fat diet-fed mice compared to those on a normal diet, with higher TSHR and FASN expression observed in the adipose tissues of obese mice [71]. These results suggest that the inhibitory effect of TSH on FASN may be canceled out by other factors that affect lipid metabolism in lipid disorder conditions. It is also possible that other molecular mechanisms are involved in the regulation of FASN by TSH, leading to increased FASN expression. Further cellular experiments and clinical studies are needed to explore the specific mechanisms underlying TSH regulation on FASN in liver cells or adipocytes under both normal and NAFLD conditions.

FASN is a key enzyme in fatty acid synthesis. Over the past decade, studies have shown that patients with subclinical hypothyroidism have higher serum levels of interleukin-6 (IL-6) and monocyte chemoattractant protein-1 (MCP–1) [72,73]. Anne Marie Gagnon *et al*. demonstrated that TSH stimulation in mouse and human adipocytes promotes lipolysis and increases serum FFA levels. These results are consistent with clinical trial results that indicated that TSH stimulates lipolysis as well as phosphorylation of lipoprotein and hormone-sensitive lipase (HSL) in a protein kinase A-dependent manner in differentiated adipocytes, leading to higher serum FFA levels in vivo [74]. A distinguishing feature of NAFLD is an increased flux of FFAs from adipose tissue [75]. Excessive uptake of FFA by hepatocytes beyond their metabolic limit induces lipotoxicity, thereby increasing the inflammatory progression of NAFLD [76].

In addition to the lipotoxic effects associated with cumulative FFAs, increased FFA levels elevate the production of reactive oxygen species (ROS), promote endoplasmic reticulum stress, and have a distinct role compared to established factors such as LDL and obesity [77]. This suggests a potential mechanism by which TSH not only increases lipid accumulation but also promotes liver fibrosis in NAFLD.

### MCP-1 and IL-6

In addition to inducing endoplasmic reticulum stress stimulated through ROS generated by FFA, TSH can indirectly promote the release of inflammatory factors to exacerbate the progression of inflammation in NAFLD.

Inflammation has been shown to play a key role in the pathogenesis of NAFLD, with some factors mediating the effects of lipids on the risk of NAFLD development [78,79]. A recent meta-analysis shows that raised IL-6 concentrations are significantly associated with an increased risk of NAFLD [80]. MCP-1, a chemokine that stimulates macrophage recruitment, is essential in the damage processes of steatohepatitis [81]. The nuclear factor kappa-B (NF-κB) signaling pathway, which is involved in inflammatory recruitment and liver injury in steatohepatitis, is a key upstream regulator of MCP-1 expression [82,83]. TSH increases the phosphorylation of protein kinase C δ (PKC-δ), which is an upstream regulator of NADPH oxidase. This takes place via TSHR, leading to the activation of the PKA and nuclear factor-kappa B kinase subunit beta (IKKβ) signaling pathways. Upon activation, PKA promotes the release of free fatty acids, while IKKβ enhances the degradation of nuclear factor-kappa B inhibitor alpha (IκB-α). Ultimately, this causes the activation of NF-κB and initiates the transcription of inflammatory cytokines, in addition to stimulating the release of the inflammatory factors IL-6 and MCP-1 [84]. The accumulation of large amounts of inflammatory factors worsens liver damage in steatohepatitis.

### SPP1

Secreted phosphoprotein-1 (SPP1), also known as osteopontin (OPN), is expressed in various organs and tissues, including adipose, liver, kidney, neurons, and macrophages. It regulates cytokine and macrophage recruitment and exacerbates chronic inflammatory responses [85–87]. Several animal studies and clinical trials have demonstrated that SPP1 mediates chronic inflammation and promotes hepatic lipid accumulation and fibrosis, thereby supporting its role in enhancing the development of NAFLD [86,88]. However, recent studies have found that the effects of SPP1 expression in different tissues or cells on NAFLD may be reversed or context-dependent. For example, SPP1 is protective against NAFLD in macrophage cells, considering that the high levels of OPN produced by macrophages (MFs) in NASH do not mediate inflammatory responses [89].

This study did not consider the effects of macrophage polarization. Macrophages exist in two polarized states, which are M1 (macrophages being pro-inflammatory) and M2 (macrophages exerting inhibitory effects). M1 macrophages secrete inflammatory cytokines and ROS, as well as stimulate the release of extracellular matrix from hepatic stellate cells (HSCs), thereby driving progressive fibrosis and the development of NAFLD [90–93]. In addition, SPP1 generates TH resistance by downregulating thyroid hormone receptor beta (TRβ), and this exacerbates lipid deposition in liver cells. The resultant TH resistance, caused by TRβ injury, leads to elevated TSH levels, which further promote the secretion of SPP1 in M1 macrophages. This establishes a positive feedback loop that accelerates NAFLD pathogenesis [94]. However, since this pathway involves thyroid hormone receptors and indirectly elevated TSH, the effects of TH cannot be completely excluded.

A growing number of studies have demonstrated that inflammation-related factors and proteins play an important role in the pathologic progression of NAFLD, with TSH potentially contributing to this process. However, the processes involved are complex, and complete independence from the influence of thyroid hormones is difficult. Consequently, there is a lack of targeted trials that focus on the overexpression and knockdown of genes to validate the relationship between TSH and SPP1, particularly concerning the specific mechanisms that involve macrophages, their polarization, and other processes linked to hepatic lipid deposition and inflammation. These areas warrant further investigation and present promising avenues for future research.

## Summary and future perspectives

The prevalence of NAFLD continues to rise annually, and its impact is becoming increasingly widespread. The roles of thyroid hormone and clinical hypothyroidism in NAFLD are well-documented. However, it has only been recently recognized that TSH impacts liver metabolism during the preclinical period. TSH modulates multiple signaling molecules that are involved in lipid metabolism, thereby contributing to lipid accumulation in the liver and the development of NAFLD. Emerging evidence suggests that TSH promotes inflammatory factors and macrophage polarization to mediate the chronic inflammatory response and hepatic fibrosis, as well as accelerate the progression of NAFLD. However, comprehensive experimental validation of these pathways remains limited, with significant overlap and interactions among molecular pathways warranting further experiments to exclude the influence of other factors. Nevertheless, existing evidence strongly supports the likelihood that TSH influences the progression of NAFLD through these mechanisms.

Currently, no thyroid hormone-based interventions for NAFLD in SCH have received approval, although numerous studies are underway. Meta-analyses across platforms like PubMed, Cochrane, and EMBASE indicate that levothyroxine (LT4) administration significantly reduces total cholesterol (TC) and LDL-C levels in SCH patients with TSH levels below 10 mIU/L. Similar benefits were even observed in patients with mild SCH. Various studies suggest that serum lipids may influence TSH regulation. Retrospective cohort studies have shown that changes in serum lipids are associated with the progression of SCH. The conclusions were consistent with previous clinical studies and animal studies: the higher the lipid levels, the higher the risk of SCH [95–97]. Further studies found that excess adipogenesis mechanisms may disrupt TSHR expression [98], suggesting a potential mechanism through which adipose-related metabolic disorders lead to an increased risk of SCH. However, studies on the reverse effect of NAFLD on hypothyroidism are limited, considering that the available data is insufficient to clarify specific mechanisms. In conclusion, the review emphasizes that although some data have limitations, numerous experimental results have demonstrated the involvement of TSH in the pathogenic mechanism of NAFLD. Therefore, TSH may be an important potential target for NAFLD prevention.

## Abbreviations

ACC: acetyl-CoA carboxylase
ACL: ATP citrate lyase
AKT: a serine/threonine-specific protein kinase
ALT: alanine aminotransferase
AMPK: AMP-activated protein kinase
BA: bile acid
BMI: body mass index
cAMP: cyclic adenosine monophosphate
Ch25h: cholesterol-25-hydroxylase
ChREBP: carbohydrate response element binding protein
CREB: cAMP-responsive element binding
CYP7A1: cholesterol 7α-hydroxylase
DNL: de novo lipogenesis
FASN: fatty acid synthase
FFA: free fatty acids
fT4: free thyroxine
HMG-CoA: 3-hydroxy-3-methylglutaryl coenzyme A
HMGCR: 3-hydroxy-3-methylglutaryl-coenzyme A reductase
HMGCS: 3-hydroxy-3-methylglutaryl-coenzyme A synthase
HNF-4α: hepatocyte nuclear factor 4 alpha
HSCs: hepatic stellate cells
HSL: hormone-sensitive lipase
IKKβ: nuclear factor-kappa B kinase subunit beta
IL-6: interleukin-6
IκB-α: nuclear factor-kappa B inhibitor alpha
LDL-c: low-density lipoprotein cholesterol
LDLR: lipoprotein receptor
LT4: levothyroxine
MCP-1: monocyte chemoattractant protein-1
MFs: macrophages
MRI: magnetic resonance imaging
mTORC: mechanistic target of rapamycin complex
NAFL: non-alcohol-associated fatty liver
NAFLD: non-alcoholic fatty liver disease
NASH: non-alcoholic steatohepatitis
NF-κB: nuclear factor kappa-B
OPN: osteopontin
PCSK9: proprotein convertase subtilisin/kexin-9
PERK: protein kinase RNA-like endoplasmic reticulum kinase
PI3K: phosphatidylinositol 3-kinase
PKA: protein kinase A
PKB: protein kinase B
PKC-δ: protein kinase C δ
rhTSH: recombinant human TSH
ROS: reactive oxygen species
SCH: subclinical hypothyroidism
SPP1: secreted phosphoprotein-1
SREBP: sterol regulatory binding protein
T3: triiodothyronine
T4: thyroxine
TC: total cholesterol
TG: triglycerides
TH: thyroid hormone
TRβ: thyroid hormone receptor beta
TSH: thyroid stimulating hormone
TSHR: thyrotropin receptor
